# Polyamine pathway inhibition as a novel therapeutic approach to treating neuroblastoma

**DOI:** 10.3389/fonc.2012.00162

**Published:** 2012-11-16

**Authors:** Laura D. Gamble, Michael D. Hogarty, Xueyuan Liu, David S. Ziegler, Glenn Marshall, Murray D. Norris, Michelle Haber

**Affiliations:** ^1^Children’s Cancer Institute Australia for Medical Research, Lowy Cancer Research CentreSydney, NSW, Australia; ^2^The Children’s Hospital of Philadelphia, Perelman School of Medicine at the University of PennsylvaniaPhiladelphia, PA, USA; ^3^Sydney Children’s HospitalSydney, NSW, Australia

**Keywords:** polyamines, MYCN, neuroblastoma, ODC1, DFMO

## Abstract

Polyamines are highly regulated essential cations that are elevated in rapidly proliferating tissues, including diverse cancers. Expression analyses in neuroblastomas suggest that up-regulation of polyamine pro-synthetic enzymes and down-regulation of catabolic enzymes is associated with poor prognosis. Polyamine sufficiency may be required for *MYCN *oncogenicity in *MYCN *amplified neuroblastoma, and targeting polyamine homeostasis may therefore provide an attractive therapeutic approach. ODC1, an oncogenic MYCN target, is rate-limiting for polyamine synthesis, and is overexpressed in many cancers including neuroblastoma. Inhibition of ODC1 by difluoromethylornithine (DFMO) decreased tumor penetrance in TH-MYCN mice treated pre-emptively, and extended survival and synergized with chemotherapy in treating established tumors in both TH-MYCN and xenograft models. Efforts to augment DFMO activity, or otherwise maximally reduce polyamine levels, are focused on antagonizing polyamine uptake or augmenting polyamine export or catabolism. Since polyamine inhibition appears to be clinically well tolerated, these approaches, particularly when combined with chemotherapy, have great potential for improving neuroblastoma outcome in both *MYCN* amplified and non-*MYCN* amplified neuroblastomas.

## INTRODUCTION

Neuroblastoma originates from the primitive cells of the sympathetic nervous system and is the most common solid tumor of early childhood. It is an aggressive cancer that often presents with high risk clinical and genetic features. In these cases, despite the use of intense multimodal therapies, long-term survival rates remain below 50% (Maris et al., 2007). Current treatment regimens are also associated with substantial morbidity, so novel therapeutic strategies are urgently needed. *MYCN *amplification, identified in up to 30% of neuroblastomas, is a powerful and reliable marker of aggressive disease and is strongly prognostic of poor outcome (Cohn and Tweddle, 2004). As a transcription factor, MYCN induces and represses a large number of genes involved in multiple biological processes including cell growth and differentiation. However, the genes necessary or sufficient to initiate neuroblastoma tumorigenesis downstream of MYCN remain to be established.

The polyamine pathway is frequently deregulated in neuroblastoma, and a number of genes involved in polyamine homeostasis are known to be MYCN or c-MYC targets (Bello-Fernandez et al., 1993; Lutz et al., 1996; Fernandez et al., 2003; Li et al., 2003; Forshell et al., 2010), while the expression of others is linked to *MYCN *status (Hogarty et al., 2008; Rounbehler et al., 2009). This suggests a mechanism by which MYCN may contribute to the malignant phenotype of neuroblastoma. Therapeutic approaches targeting the polyamine pathway may therefore provide an effective strategy for the treatment of high risk neuroblastoma, particularly in tumors dependent on deregulated Myc activity, such as those with *MYCN *amplification.

## REGULATION OF THE POLYAMINE PATHWAY

**FIGURE 1:**
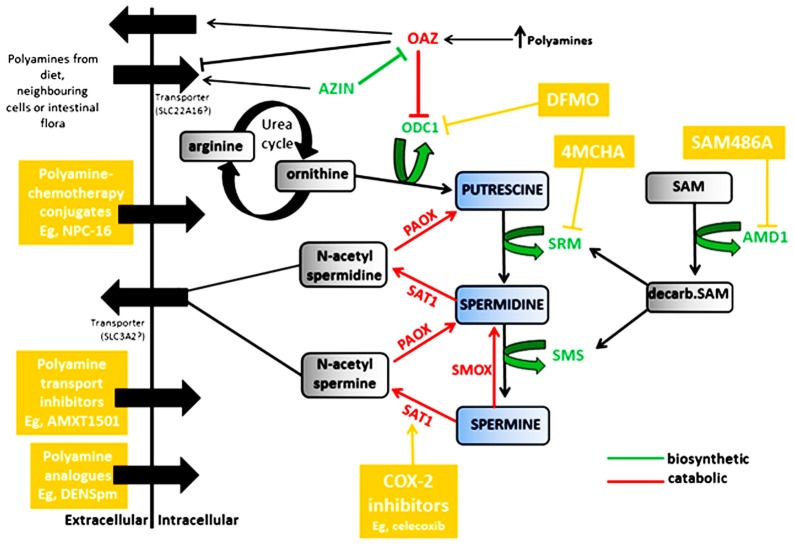
**Regulation of the polyamines putrescine, spermidine and spermine by biosynthetic enzymes (shown in green) and catabolic enzymes (shown in red)**. Compounds and classes of compounds that target various aspects of polyamine regulation are shown in yellow. ODC1, ornithine decarboxylase; OAZ, antizyme; AZIN, antizyme inhibitor; SRM, spermidine synthase; SMS, spermine synthase; AMD1, adenosylmethionine decarboxylase; SAT1, spermine/spermidine N_1_-acetyltransferase; PAOX, polyamine oxidase; SMOX, spermine oxidase.

Polyamines are positively charged multifunctional polycations derived from amino acids and found in all living organisms. They are indispensable for cell growth, differentiation, and cell survival and function by forming electrostatic bonds with negatively charged macromolecules to mediate a number of biological processes. These include DNA synthesis and stability, replication, transcription and translation, ribosome biogenesis, modulation of ion channels and receptors, and protein phosphorylation (Pegg, 1988; Panagiotidis et al., 1995; Johnson, 1996; Igarashi and Kashiwagi, 2000; Childs et al., 2003; Gerner and Meyskens, 2004; Pegg, 2006). Polyamines are also required for covalent activation of eIF5A, a major protein translation factor, via hypusination, a polyamine-dependent modification (Cooper et al., 1983). Whereas polyamine depletion leads to growth arrest, overexpression of these essential cations is cytotoxic (Poulin et al., 1993; Tobias and Kahana, 1995; Ray et al., 2001; Li et al., 2002). Therefore, tight regulation of intracellular polyamine levels is critical and is dependent on the proliferative state of the cell. Regulatory mechanisms include *de novo* synthesis, recycling via a back converting catabolic pathway and through transmembrane import and efflux (Gerner and Meyskens, 2004; Casero and Marton, 2007). An overview of the polyamine pathway is shown in **Figure 1**.

### POLYAMINE BIOSYNTHESIS

The first rate-limiting enzyme in the polyamine pathway is ornithine decarboxylase (ODC1), which catalyzes the decarboxylation and conversion of ornithine, a product of the urea cycle, to the primary polyamine putrescine (Pegg, 2006). Putrescine is the precursor for spermidine and spermine synthesis, and is further processed into these more abundant polyamines by two aminopropyltransferases, spermidine synthase (SRM) and spermine synthase (SMS). The second rate-limiting enzyme, adenosylmethionine decarboxylase (AMD1), decarboxylates S-adenosylmethionine (SAM) to provide the aminopropyl donor for the conversions to spermidine and spermine. Both ODC1 and AMD1 are highly controlled at the transcriptional and post-transcriptional levels, and have among the shortest half-lives of any mammalian enzymes. In addition, ODC1 turnover is regulated by antizymes (OAZ1, OAZ2, and OAZ3) which in turn are controlled by antizyme inhibitors (AZIN1 and AZIN2). Antizymes initiate ODC1 degradation by binding the ODC monomer, inhibiting its activity and shunting ODC1 to the 26S proteasome for degradation (Li and Coffino, 1992; Murakami et al., 1992). Of the three antizymes, OAZ1 is the most effective at stimulating ODC1 degradation. Antizyme expression is also induced by a feedback mechanism. An increase in intracellular polyamine levels stimulates a +1 frame-shift by the ribosomes during translation of antizyme mRNA, increasing expression of the full-length protein (Matsufuji et al., 1995). In response to increased intracellular polyamines, antizymes negatively regulate polyamine transport by promoting polyamine secretion and inhibiting uptake, while antizyme degradation by the ubiquitin pathway is also inhibited (Mitchell et al., 1994; Suzuki et al., 1994; Palanimurugan et al., 2004).

Antizyme inhibitors antagonize the function of antizymes by mimicking ODC1 (Koguchi et al., 1997; Kanerva et al., 2008). They are highly homologous to ODC1, but lack enzymatic activity due to critical amino acid substitutions and bind antizymes with greater affinity than ODC1 (Albeck et al., 2008). Increased antizyme inhibitor activity therefore results in the release of ODC1 from the inactive ODC1-antizyme complex, which in turn increases the production of polyamines (Murakami et al., 1996; Mangold, 2006; Pegg, 2006). In addition, forced induction of AZIN1 in cell cultures has also been shown to increase polyamine uptake (Keren-Paz et al., 2006). Polyamine levels themselves act as down-regulators of both ODC1 and AMD1 and as up-regulators of antizymes by a feedback homeostasis mechanism.

### POLYAMINE CATABOLISM

Polyamine catabolism allows for the re-utilization of polyamines as spermine is converted back to spermidine and spermidine back to putrescine. A number of key enzymes are involved in this process as shown in **Figure 1**. The degradation of polyamines depends on three enzymes; spermine/spermidine N_1_-acetyltransferase (SAT1), polyamine oxidase (PAOX), and spermine oxidase (SMOX). SAT1, a highly inducible cytosolic enzyme, acetylates spermine and spermidine (Casero and Pegg, 1993), which are then either exported from the cell, or oxidized by the peroxisomal enzyme PAOX, resulting in conversion to spermidine or putrescine, H_2_O_2_ and 3-aminopropanol (Seiler, 1995). PAOX preferentially catalyzes the oxidation of the N_1_-acetylspermine/spermidine produced by SAT1 activity, rather than spermine or spermidine, whereas SMOX is a cytosolic enzyme which catalyzes the oxidation of spermine directly to spermidine, without acetylation and produces H_2_O_2_ and 2 aminopropanol (Vujcic et al., 2002; Wang et al., 2003; Casero and Pegg, 2009; Pegg, 2009). Mostly, PAOX is constitutively expressed and dependent on SAT1 as it is rate-limited by the availability of the acetylated spermidine/spermine (Casero and Pegg, 1993; Vujcic et al., 2002). SAT1, the rate limiting enzyme in polyamine catabolism, is therefore extensively regulated at transcriptional and post-transcriptional levels (Fogel-Petrovic et al., 1993; Coleman et al., 1996), and is a gatekeeper regulating flux through the polyamine pathway (Kramer et al., 2008).

### TRANSMEMBRANE IMPORT AND EFFLUX

Cellular polyamine levels are also regulated by transmembrane transport where cells can take up polyamines from their surroundings and also export them to the extracellular space, and this can make a significant contribution to cellular polyamine levels. Known polyamine transporters include SLC3A2 (Uemura et al., 2008) and SLC22A16 (Aouida et al., 2010). SAT1 is co-localized with the SLC3A2 transporter and catalyzes the export of acetylated polyamines via a polyamine/arginine exchange reaction, suggesting a role for acetylation in polyamine efflux (Uemura et al., 2008). SLC22A16 has also been identified as a high affinity transporter directing polyamine import in mammalian cells (Aouida et al., 2010). Polyamine uptake by caveolae-dependent endocytosis has also been reported (Roy et al., 2008). Polyamines are present in the extracellular space from dietary intake, export from neighboring cells and synthesis by intestinal bacteria. Such microenvironment polyamines provide a reservoir whereby polyamine antagonized cancer cells can circumvent biosynthetic blockade through augmented uptake.

## ABERRANT EXPRESSION WITHIN THE POLYAMINE PATHWAY IN NEUROBLASTOMA, AND THE ASSOCIATION WITH MYCN

Polyamines are elevated in rapidly proliferating cells, including cancer cells, and substantial evidence suggests cancer development is associated with altered polyamine regulation. The biological association between increased polyamines and tumor formation is well established in numerous cancers including breast, prostate, colon, skin carcinoma and neuroblastoma (Cipolla et al., 1993; Leveque et al., 2000; Thomas and Thomas, 2003; Gerner and Meyskens, 2004; Casero and Marton, 2007). There is also evidence that increased polyamine biosynthesis is not just a consequence of increased proliferation in these cells, but may be necessary for the development of specific cancers (Gerner and Meyskens, 2004; Casero and Marton, 2007). The mechanism by which *MYCN* amplification results in such a poor prognosis has yet to be fully elucidated, and recent evidence suggests that its effect on the polyamine pathway may play a critical role. A number of polyamine genes have been shown to be c-MYC target genes (*ODC1*, *AMD1*, and *SRM*) whereas others appear to be regulated by MYC/MYCN (Bello-Fernandez et al., 1993; Fernandez et al., 2003; Hogarty et al., 2008; Rounbehler et al., 2009; Forshell et al., 2010). However, with the exception of *ODC1 *(Lutz et al., 1996), the polyamine genes that are direct transcriptional targets of MYCN remain to be established. It is highly likely that polyamine synthesis may be specifically required to support downstream MYCN-governed functions.

*ODC1* is a well-established oncogene in its own right (Auvinen et al., 1992), with high ODC1 activity associated with tumor growth in several human cancers, including neuroblastoma (O’Brien et al., 1975; Janne et al., 1978; Scalabrino and Ferioli, 1981; Crozat et al., 1992; Mohan et al., 1999; Wallace and Caslake, 2001; Hogarty et al., 2008). The contribution of ODC1 activity to MYC-induced lymphomagenesis was examined in a mouse model of B-cell lymphoma, the *Eμ*-*Myc* transgenic mouse. In this model, ODC1 ablation inhibited lymphomagenesis, but subsequent restoration of ODC1 activity promoted tumor onset (Nilsson et al., 2005). In addition, enforced expression of ODC1 in the skin of transgenic mice led to increased tumor incidence (O’Brien et al., 1997; Chen et al., 2000). In neuroblastoma there is significant evidence that ODC1 is overexpressed in high risk disease. It is often co-amplified with *MYCN* or overexpressed, and is associated with poor prognosis in both *MYCN* amplified and non-*MYCN* amplified tumors (Hogarty et al., 2008; Rounbehler et al., 2009; Geerts et al., 2010).

**FIGURE 2:**
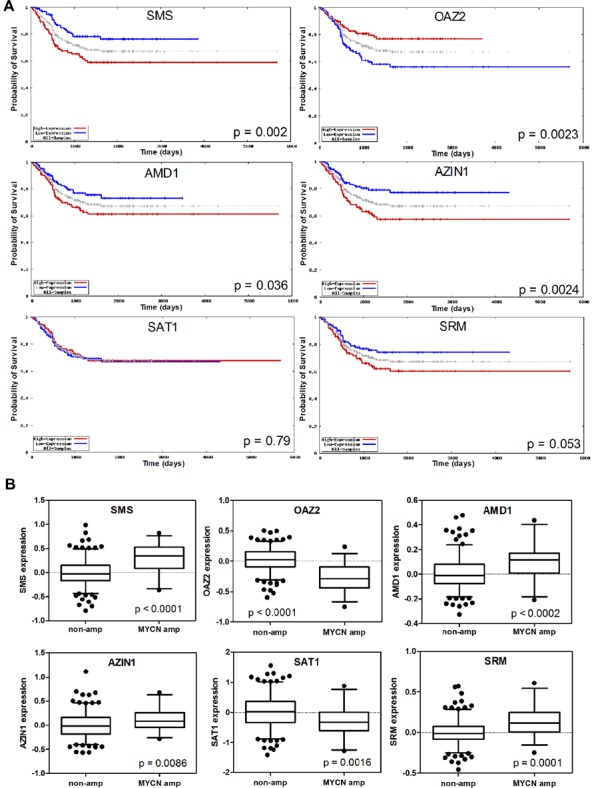
**Analysis of expression of the polyamine pathway regulators SMS, OAZ2, AMD1, AZIN1, SAT1, and SRM, and their association with neuroblastoma outcome**. **(A)** Kaplan–Meier survival curves in the overall neuroblastoma cohort with dichotomization for high/low expression around the median. **(B)** Expression of polyamine pathway genes in the subsets of tumors with and without *MYCN* amplification. Data was obtained from the Neuroblastoma Prognosis Database (publically available at http://home.ccr.cancer.gov/oncology/oncogenomics/).

Evaluation of several polyamine genes included in the Neuroblastoma Prognosis Database (publically available at http://home.ccr.cancer.gov/oncology/oncogenomics/) revealed that increased expression of biosynthetic *SMS*, *AMD1*, and *AZIN*, and decreased expression of catabolic *OAZ2* was associated with decreased survival and poor prognosis as shown in **Figure 2A**. The levels of *SAT1 *or *SRM *expression on the other hand, were not prognostic of survival. However, all of these genes, including *SAT1* and *SRM*, were associated either positively or negatively with *MYCN *amplification dependent on their biosynthetic or catabolic role (**Figure 2B**). Since MYCN is upstream of the polyamine biosynthesis pathway, this suggests a major role for MYCN in regulating polyamine biosynthesis, and a mechanism by which MYCN contributes to neuroblastoma development. Several studies support these findings. Geerts et al. (2010) found increased *ODC1* and reduced *OAZ2 *expression to be excellent predictors of survival and poor prognosis in both *MYCN* amplified and non-amplified neuroblastomas. OAZ1 and OAZ3 on the other hand played no role in predicting survival. Transcriptome analysis of 101 primary neuroblastomas found several polyamine biosynthetic genes, including *ODC1*, *AMD1*, *SRM*, and *SMS*, to be up-regulated in the *MYCN* amplified high risk cohort (and again *ODC1* expression was elevated in non-*MYCN* amplified high risk group; Hogarty et al., 2008). *OAZ2* was expressed at lower levels in high risk *MYCN *amplified tumors but also significantly reduced in non-*MYCN* amplified high risk tumors. In addition catabolic *SMOX *was decreased, while the level of *SAT1* expression was not associated with any particular risk group (Hogarty et al., 2008). These studies suggest a role for ODC1, and OAZ2, independent of MYCN, in promoting an aggressive phenotype. Further evidence supporting this conclusion comes from the finding that *ODC1* is not always co-amplified with *MYCN* in neuroblastomas, while copy number gain of *ODC1 *has been reported in half of high risk neuroblastomas without *MYCN* amplification, suggesting a mechanism by which the polyamine pathway is up-regulated in this subset (George et al., 1997; Mosse et al., 2007; Hogarty et al., 2008).

These data suggest that systemic alterations in polyamine metabolism correlate with *MYCN* amplification, but that polyamine enhancement in non-*MYCN* amplified tumors is also associated with high risk disease. Polyamine depletion strategies may be broadly effective against high risk tumors, rather than just *MYCN *amplified tumors.

## TARGETING POLYAMINE BIOSYNTHESIS AS A THERAPEUTIC APPROACH IN NEUROBLASTOMA

Since elevated polyamines are sustained in rapidly proliferating cells and levels are increased in cancer tissues compared to surrounding tissues, suppression of polyamine biosynthesis provides an attractive therapeutic approach for many cancers. Inhibitors of the rate-limiting enzymes in polyamine biosynthesis, ODC1 and AMD1, have been developed and extensively tested in preclinical and clinical trials. α-difluoromethylornithine (DFMO) acts as a specific suicide inhibitor of ODC1 and is the most widely studied inhibitor of polyamine metabolism both as a chemotherapeutic and a chemopreventive agent (Meyskens and Gerner, 1999; Levin et al., 2000; Takahashi et al., 2000; Fabian et al., 2002; Levin et al., 2003). Exposure of a number of cancer cell lines, tumors and tissues to DFMO has shown a considerable decrease in intracellular putrescine concentrations, subsequent decreases in spermidine levels, and growth inhibition as a result of impaired synthesis of RNA, DNA, and proteins (Mamont et al., 1982; Sunkara and Rosenberger, 1987). Despite promising preclinical results, the anti-tumor activity of DFMO has to date failed to translate to the clinic. However, further investigations have shown additive and synergistic activities when used in combination therapies for the treatment of specific cancers in animal models (Bartholeyns and Koch-Weser, 1981; Marton, 1987; Quemener et al., 1992) and the potential for synthetic-lethal interactions in MYC-driven cancers specifically has yet to be directly tested.

It has previously been shown that ODC1 heterozygosity or treatment with DFMO impairs MYC induced lymphomagenesis in the *Eμ-Myc* transgenic mice, and that this was achieved though impairment of MYC’s ability to suppress p27^KIP1^, a CDK inhibitor (Nilsson et al., 2005). A number of recent studies have investigated the effect of DFMO in neuroblastoma cell lines and animal models. A *MYCN* transgenic mouse model (TH-MYCN mice), which faithfully recapitulates the features of human neuroblastoma with similar biochemical features and syntenic chromosomal rearrangements to human neuroblastoma, was used (Weiss et al., 1997). Tumor formation is dependent on the level of *MYCN* gene dosage, with homozygous mice developing tumors with a short latency and 100% tumor penetrance, and hemizygous mice displaying longer tumor latency and only 20–30% penetrance. In this model TH-MYCN tumors have up-regulated *ODC1*, *AZIN*, *AMD1*, *SRM*, and *SMS*, and down-regulated *OAZ2*, *SMOX*, and *SAT1* compared with sympathetic ganglia (Evageliou and Hogarty, 2009). Since a similar pattern of polyamine deregulation is seen in primary human neuroblastoma, this suggests that results using this model are likely to be translatable to the human disease. DFMO treatment in neuroblastoma cell lines inhibited proliferation, and when extended to *in vivo* studies using the TH-MYCN transgenic mouse, DFMO treatment from birth increased tumor latency and overall survival (Hogarty et al., 2008). Interestingly, no tumors developed after DFMO withdrawal, suggesting a finite period during which embryonic tumors can develop and also supporting a role for DFMO as a chemopreventive agent for neuroblastoma. Giving hemizygous mice DFMO from birth resulted in reduced tumor initiation. DFMO treatment of mice with detectable tumors delayed tumor progression and extended survival time (Hogarty et al., 2008). Similarly, Rounbehler et al. (2009) found that DFMO selectively impaired the proliferative response of *MYCN* amplified neuroblastomas, and delayed tumor incidence and onset *in vivo*. However, once a tumor developed it had a similar aggressive phenotype as tumours in mice that had not received DFMO, and whilst there was a reduction in putrescine, spermidine was only moderately reduced and spermine levels remained unchanged (Hogarty et al., 2008). Importantly, DFMO enhanced the effect of the anticancer drugs, cyclophosphamide and cisplatin *in vivo. *Tumor-free survival after cyclophosphamide treatment in combination with DFMO was increased to 80% compared to 20% for cyclophosphamide alone, and DFMO significantly increased the survival time of mice treated with cisplatin, although all of these mice did eventually succumb to the disease (Hogarty et al., 2008).

SAM486A is a derivative of the first generation AMD1 inhibitor mitoguazone (MGBG), and exerts potent and specific inhibition of AMD1 (Regenass et al., 1992, 1994). Its efficacy has been assessed in a number of cancer cells and animal systems, and has been tested in phase I and II clinical trials in adult cancers. However, like DFMO, when used as a single agent, results have been disappointing. In neuroblastoma, *in vitro *studies found p53 wild-type cells to be highly sensitive to SAM486A independent of their *MYCN* status (Koomoa et al., 2009). In these cells SAM486A functions by inducing p53, possibly through DNA damage induced by ATM, and by reducing Akt/PKB expression to induce apoptosis and inhibit cell proliferation (Koomoa et al., 2009). In addition, large increases in intracellular putrescine levels correlated with increased p53. SAM486A treatment of p53 mutant neuroblastoma cells inhibited polyamine-dependent cell growth and caused a G_1_ arrest, which was further enhanced upon combination with DFMO. Neither compound, either alone or in combination, induced apoptosis (Wallick et al., 2005). Following removal of these inhibitors in the p53 mutant cells, the proliferative capacity of the cells was slow and only partially restored, but this was shown to be largely due to DFMO and not SAM486A. DFMO has been shown to induce cell cycle arrest in a p53 mutant neuroblastoma cell line via induction of two contradictory pathways; cell survival via PI3K/PKB signaling, and cell cycle arrest through p27^KIP1^ phosphorylation (Wallick et al., 2005; Koomoa et al., 2008).

The disappointing clinical trials with either DFMO or SAM486A as single agents are likely due to activation of compensatory mechanisms following DFMO or SAM486A exposure. This allows intracellular polyamine levels to be maintained in a cell upon loss of a single biosynthetic enzyme activity. Polyamines may be imported from extracellular pools, and compensatory induction of other biosynthetic enzymes or reduced polyamine catabolism may be involved.

AMD1 has been shown to be up-regulated following ODC1 inhibition (Wallick et al., 2005) and it has been reported that combined DFMO and SAM486A therapy is synergistic in neuroblastoma (Evageliou and Hogarty, 2009). This indicates that attacking the polyamine synthesis pathway with multiple compounds may be a more effective approach, particularly if the two rate-limiting enzymes are simultaneously inhibited. DFMO and SAM486A are of particular interest because clinical trials in other cancer types have shown both inhibitors to be well tolerated, even at high doses, with only the occasional occurrence of reversible ototoxicity (DFMO only), nausea and mild neutropenia. In addition, DFMO is already FDA approved as it is used in the treatment of African trypanosomiasis (Van Nieuwenhove et al., 1985; Sjoerdsma and Schechter, 1999).

As well as being tested as a chemotherapeutic agent, DFMO has demonstrated promising results in human trials as a chemopreventive agent. This polyamine inhibitor has been shown to suppress skin carcinogenesis in patients with moderate to severe actinic keratosis (Alberts et al., 2000), and also slowed prostate cancer growth in men with a family history of prostate cancer (Simoneau et al., 2008). In addition, DFMO in combination with sulindac, a SAT1 inducing COX2 inhibitor, resulted in a remarkable decrease in colon adenomas in patients with previous disease (Meyskens et al., 2008). There were no significant toxicities in any of these studies. Whilst chemopreventive approaches are not currently practical in neuroblastoma, the use of polyamine antagonists could prove useful in managing minimal residual disease (MRD) post-autologous stem cell transplantation in order to reduce the risk of relapse.

Another compound that targets polyamine biosynthesis is the SRM inhibitor, trans-4-methylcyclohexamide (4MCHA). This inhibitor has been tested in a B-cell lymphoma mouse model where 4MCHA had chemopreventive effects *in vivo*, but was not effective against established lymphomas (Shirahata et al., 1993; Forshell et al., 2010). SRM is a MYC target and interestingly, it was found to be more potently induced by MYC than ODC1 suggesting it may be important in MYC-induced oncogenesis.

## OTHER MECHANISMS OF POLYAMINE DEPLETION

### POLYAMINE ANALOGS AND INDUCTION OF POLYAMINE CATABOLISM

Polyamine analogs have a multistep role in depleting polyamine pools. They function by mimicking natural polyamines and subsequently lowering intracellular polyamine levels by feedback inhibition. The result is down-regulation of synthetic enzymes such as ODC1 and AMD1, but also induction of catabolic enzymes such as SAT1 and SMOX. Elevated levels of SAT1 increases export of acetyl-polyamines due to co-localization with a polyamine transporter (such as SLC3A2), and induction of SMOX results in a subsequent production of H_2_O_2_ (Wang et al., 2001; Casero et al., 2003; Pledgie-Tracy et al., 2009). The resulting accumulation of non-functional analogs competitively inhibits polyamine function, and the depleted intracellular polyamine pool reduces cell proliferation and induces growth inhibition (Reddy et al., 1998; Casero and Marton, 2007). A number of these compounds have been developed and have shown promising results in *in vitro* and *in vivo* models.

N^1^, N^11^-diethylnorspermine (DENSpm) is a spermine analog that induces growth inhibition in a number of cancer cell lines (Kramer et al., 1997; Gabrielson et al., 1999; Schipper et al., 2000; Myhre et al., 2008). When tested in neuroblastoma cell lines, the compound induced G_1_ arrest and apoptosis in p53 wild-type cells, but similar to SAM486A treatment, only induced growth inhibition in p53 mutant cells (Soderstjerna et al., 2010). DENSpm, as well as the related compound, BENSpm, and the second-generation CPENSpm, have all been tested in phase I and II clinical studies but were not successful as single agents (Creaven et al., 1997; Hahm et al., 2002; Wolff et al., 2003). BENSpm has been shown to synergistically induce growth inhibition in combination with standard chemotherapy agents in cell lines (Davidson et al., 1993; Pledgie-Tracy et al., 2009). In addition BENSpm combined with cisplatin produced synergistic cell death in cisplatin resistant ovarian carcinoma cells (Marverti et al., 1998). This resulted in a synergistic increase in SAT1 activity, and the subsequent polyamine pool depletion by this combination was significantly greater than either agent alone (Tummala et al., 2011). However*, in vivo* studies using breast cancer cell xenografts found that BENSpm in combination with paclitaxel did not further reduce tumor growth compared to either agent alone. *In vitro*, synergy between BENSpm and various chemotherapeutic agents was dependent on the cell line and the combined chemotherapeutic used (Pledgie-Tracy et al., 2009). Surprisingly, whereas some studies report induction of SMOX by polyamine analogs, BENSpm and CPENSpm have been shown to bind the catalytic site of SMOX, inhibiting activity and may explain why SMOX levels appear to be induced, as a result of stabilization (Cervelli et al., 2010). The subsequent reduction in H_2_O_2_ in the tumor mass may partly explain the lack of activity observed in clinical trials.

PG11047 is a novel conformationally restricted analog of spermine that competitively inhibits spermine function (Reddy et al., 1998), and has recently been studied in phase I clinical trials for adult cancers both as a single agent and in combination with chemotherapy (Clinical Trial Identifier: NCT00705874). PG11047 inhibits cell proliferation in a range of cell lines, and inhibits tumor growth *in vivo* in prostate and NSCLC xenografts (Hacker et al., 2008; Dredge et al., 2009). However, whilst Ewings sarcoma cell lines were particularly sensitive to PG11047-mediated growth inhibition, neuroblastoma cells were less sensitive and *in vivo*, a cytostatic effect similar to that seen with DFMO was reported, with only a modest delay in tumor onset after treatment (Smith et al., 2011).

### INHIBITION OF POLYAMINE UPTAKE

Since tumor cells exhibit enhanced polyamine transport activity by comparison with normal cells, and since the pharmacological inhibition of polyamine biosynthesis leads to a compensatory increase in polyamine salvaging activity (Pegg, 1988; Seiler et al., 1996), another mechanism of inhibiting this pathway includes antagonizing polyamine uptake. A number of compounds are under preclinical development, including D-lysine spermine (MQT-1426), N1-spermyl-L-lysinamide (OR1202), and a spermine analog D-Lys(C_16_acyl)-Spm (AMXT1501; Weeks et al., 2000; Chen et al., 2006; Burns et al., 2009). Despite a poor response to D-Lys(C_16_acyl)-Spm alone, combination with DFMO had remarkable efficacy against cutaneous squamous cell carcinomas (SCC) in a transgenic *ODC1* mouse model of skin cancer. The majority of large aggressive SCCs underwent complete or near-complete remission, even in the presence of extracellular spermidine, indicating that D-Lys(C_16_acyl)-Spm in combination with DFMO is successful in reducing intracellular polyamines (Burns et al., 2009). Available evidence indicates that high levels of expression of SLC22A16, a polyamine transporter, are prognostic of poor outcome in *MYCN* non-amplified metastatic neuroblastoma (http://pob.abcc.ncifcrf.gov/cgi-bin/JK). Targeting this polyamine transporter may be required to effectively reduce intracellular polyamine levels.

The use of NSAIDs such as celecoxib and sulindac, has also been investigated, which function by influencing polyamine acetylation and export through up-regulation of SAT1 (Babbar et al., 2003). Celecoxib combined with anticancer agents induces synergistic and anti-proliferative effects (Shirode and Sylvester, 2010), exerting their chemopreventive action by affecting SAT1. DFMO in combination with NSAIDs has been shown to suppress colorectal carcinogenesis in murine models and in phase II clinical trials (Fischer et al., 2003; Gerner et al., 2007).

### POLYAMINE-CHEMOTHERAPY CONJUGATES

Polyamines conjugated to cytotoxic drugs, such as naphthalimide, anthracene, or anthraquinone, can be transported into cancer cells via the polyamine transporter system, and have been shown to exert potent anti-tumor effects (Tian et al., 2009; Xie et al., 2011a). Since the polyamine transporter is up-regulated in many tumor cells, these compounds may provide a targeted therapy, inhibiting cell proliferation through simultaneously delivering a cytotoxic drug, and also depleting intracellular polyamine content. A number of preclinical studies have shown promising results in a variety of cancers, although no studies have been carried out in neuroblastoma. In colorectal cancer cell lines, a naphthalimide-polyamine conjugate (NPC-16) in combination with celecoxib produced enhanced apoptosis as a result of elevated NPC-16 uptake due to up-regulated SAT1 activity, and decreased intracellular polyamine levels (Xie et al., 2011b). A putrescine conjugated with anthracene, Ant 4, was shown to induce cytotoxicity and subsequent apoptosis in a promyelogenous leukemia cell line (Palmer et al., 2009). Putrescine uptake was significantly reduced, demonstrating that this conjugate could successfully compete with its native polyamine for uptake. The spermine-podophyllotoxin conjugate F14512 has shown exceptional cytotoxicity in cells with enhanced polyamine uptake *in vitro*, as well as inhibiting breast carcinoma in a xenograft model (Barret et al., 2008). Whilst preclinical data using this class of compound look promising, to date no clinical trials have taken place. It is attractive to speculate that combining a polyamine-chemotherapy conjugate with other polyamine depleting agents will facilitate the uptake of these conjugates and provide a more active targeted approach in reducing polyamines.

## CONCLUSION

Many compounds targeting the polyamine pathway have been developed or are under development. However, those that have made it to clinical trials have produced limited effects, most likely as a result of compensatory mechanisms that allow a cell to circumvent polyamine depletion. Polyamine depletion compounds have been well tolerated clinically, and in combination with chemotherapeutic agents have produced synergistic effects. It is likely that optimization of polyamine depletion, by using compounds that decrease polyamine synthesis and eradicate compensatory mechanisms, in combination with chemotherapeutic agents may have significant clinical potential in improving the outcome of patients with aggressive neuroblastoma. Furthermore, these compounds are likely to be effective in both *MYCN* amplified and non-*MYCN* amplified patients since polyamine deregulation has been observed in both tumor groups. A phase I clinical trial, coordinated by the New Approaches to Neuroblastoma Therapy (NANT) consortium, for the treatment of refractory neuroblastoma using high dose DFMO and celecoxib in combination with standard chemotherapy (cyclophosphamide and topotecan) is in development, and results from this study will be invaluable in determining the potential use of polyamine depletion for the treatment of neuroblastoma.

## Conflict of Interest Statement

The authors declare that the research was conducted in the absence of any commercial or financial relationships that could be construed as a potential conflict of interest.
